# Overview of Zika virus (ZIKV) infection in regards to the Brazilian epidemic

**DOI:** 10.1590/1414-431X20165420

**Published:** 2016-04-29

**Authors:** S.N. Slavov, K.K. Otaguiri, S. Kashima, D.T. Covas

**Affiliations:** 1 Hemocentro de Ribeirão Preto, Faculdade de Medicina de Ribeirão Preto, Universidade de São Paulo, Ribeirão Preto, SP, Brasil; 2 Departamento de Clínica Médica, Faculdade de Medicina de Ribeirão Preto, Universidade de São Paulo, Ribeirão Preto, SP, Brasil; 3 Departamento de Análises Clínicas, Toxicológicas e Bromatológicas, Faculdade de Ciências Farmacêuticas, Universidade de São Paulo, Ribeirão Preto, SP, Brasil

**Keywords:** Zika virus, ZIKV, Transmission, Epidemiology, Congenital infection, Brazil

## Abstract

Zika virus (ZIKV), a mosquito-borne flavivirus, belongs to the *Flaviviridae* family, genus *Flavivirus*. ZIKV was initially isolated in 1947 from a sentinel monkey in the Zika forest, Uganda. Little clinical importance was attributed to ZIKV, once only few symptomatic cases were reported in some African and Southeast Asiatic countries. This situation changed in 2007, when a large outbreak was registered on the Yap Island, Micronesia, caused by the Asian ZIKV lineage. Between 2013 and 2014, ZIKV spread explosively and caused many outbreaks in different islands of the Southern Pacific Ocean and in 2015 autochthonous transmission was reported in Brazil. Currently, Brazil is the country with the highest number of ZIKV-positive cases in Latin America. Moreover, for the first time after the discovery of ZIKV, the Brazilian scientists are studying the possibility for the virus to cause severe congenital infection related to microcephaly and serious birth defects due to the time-spatial coincidence of the alarming increase of newborns with microcephaly and the Brazilian ZIKV epidemic. The present review summarizes recent information for ZIKV epidemiology, clinical picture, transmission, diagnosis and the consequences of this emerging virus in Brazil.

## Introduction

**Figure 1:**
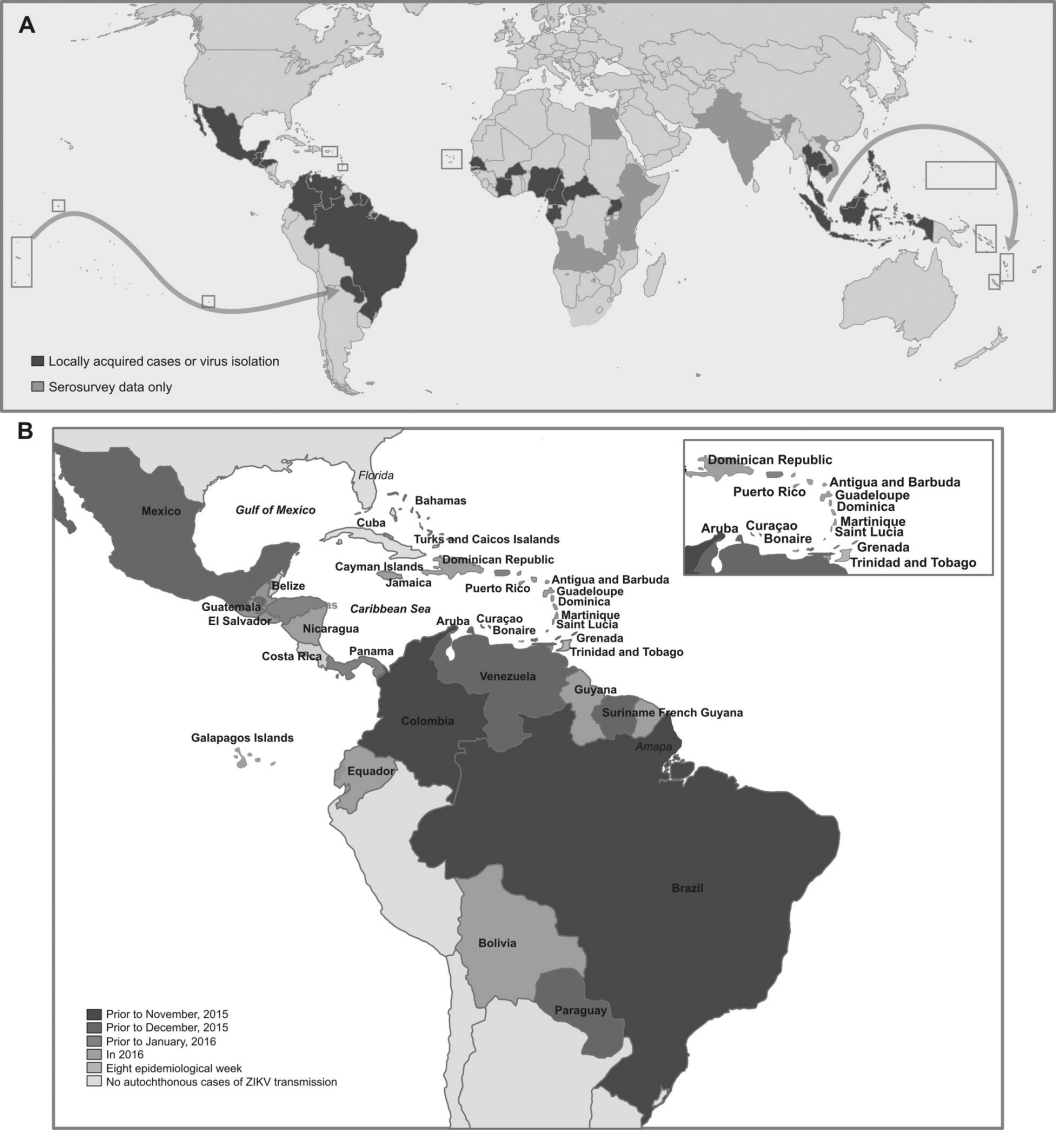
World epidemiology of ZIKV infection. *A*, Global expansion of the Asian lineage. After the characterization and isolation of ZIKV in Uganda, until 2007 only few human cases were reported in some African and Southeast Asiatic countries. The first significant ZIKV outbreak was registered in Yap Island, Micronesia, geographically situated close to Indonesia, where ZIKV is endemic (arrow). Between 2013 and 2014, ZIKV was rapidly spread to many of the islands of the Southern Pacific Ocean, causing the largest outbreak in French Polynesia. In 2015, the first autochthonous cases were detected in Brazil. *B*, Epidemiology of ZIKV infection in Central and South America. Currently, Brazil is the most affected country in South America followed by Colombia (maps adapted from the Pan-American World Health Organization, PAHO, Zika-epidemiological update).

Zika virus (ZIKV) was isolated in 1947 from a febrile sentinel *Rhesus* monkey from the Zika forest on the banks of lake Victoria, Uganda (1). The virus was not linked to a human infection until 1954, when ZIKV was detected in three patients during an outbreak of jaundice in East Nigeria (2). Almost at the same time, ZIKV has been isolated from *Aedes (Stegomya) africanus* mosquitoes, in Africa, and *Aedes aegypti*, in Southeast Asia (3,4). From 1954 to 1993 serologic evidence for ZIKV infection had been reported from various African countries including Kenya (5), Sierra Leone (6), Gabon (7,8), Ivory Coast (9), Central African Republic (10), and Senegal (11). By this time, in Asia, ZIKV has been detected in individuals with acute fever from Central Java and Lombok Island, Indonesia (12,13). This early serological evidence demonstrated that ZIKV circulates in some countries of Sub-Saharan Africa and Southeast Asia with relatively low number of symptomatic cases (<10 official cases). The situation changed in 2007, when an outbreak of a Dengue virus (DENV)-like disease characterized by rash, conjunctivitis and arthralgia was registered on the Yap Island, Micronesia (Figure 1). Rapid DENV tests suggested that it was the etiological agent for that outbreak and the collected samples were sent to the Centers for Disease Control and Prevention (CDC) Arbovirus Diagnostic Laboratory for viral confirmation. The performed tests with *Flaviviridae* genus consensus primers demonstrated sequences sharing 90% nucleotide identity with ZIKV. Therefore, the Yap Island epidemic was caused by ZIKV and not DENV and, specifically, by the ZIKV Asian lineage (14). The Yap Island outbreak demonstrated that ZIKV was not restricted to Africa and Southeast Asia and could cause epidemics outside its habitual occurrence. Nevertheless, Micronesia is geographically close to the countries of Southeast Asia, where ZIKV is endemic and it could be supposed that travelers or trade have introduced the virus in Oceania. Surprisingly, between 2013 and 2014, ZIKV continued its spread through the Southern Pacific and caused its largest outbreak in French Polynesia with an estimated occurrence of 19,000 cases in various islands from this region. The identified genotype, similarly to the epidemic in the Yap Island, belonged to the Asiatic lineage and its global expansion was suspected (15). Moreover, the Asian lineage expanded rapidly to many South Pacific islands and outbreaks were reported from New Caledonia, the Cook Islands and the Easter Island, Chile (16) (Figure 1).

The first official report for ZIKV autochthonous transmission in Brazil was documented in March 2015 in the city of Natal, in the Northeast part of the country. The patients presented with a benign disease characterized by mild fever, macopapular rash, headache, conjunctivitis, arthralgia and edema, similar to the symptoms caused by DENV, however all were DENV-negative (17). After confirmation of autochthonous transmission of ZIKV in Brazil, it was speculated that ZIKV entered the country during the 2014 World Football Championship; however, no countries endemic for ZIKV infection were competing in this event. The most probable ZIKV introduction in Brazil seems to have occurred during the Va’a World Sprint Championship, where competitors from countries with ZIKV outbreaks, i.e., French Polynesia, New Caledonia, Cook Islands, and Easter Island, have been participating (18). This event coincided with the first report of autochthonous transmission of ZIKV in Brazil and with the confirmation of the Asiatic lineage of the circulating strain. Currently, Brazil is the country with the highest number of ZIKV-positive cases in Latin America with circulation of the virus in almost the entire national territory.

### Viral characteristics

ZIKV belongs to the *Flaviviridae* family, genus *Flavivirus*, closely related to the *Spondweni* virus (19). Therefore, ZIKV has the typical flavivirus organization of the virion with an icosahedral, enveloped particle. The genome is a single-stranded, positive-sense RNA of approximately 11 kb. The genomic organization follows that of the flaviviruses with two flanking non-coding regions (NCR), i.e. 59 and 39, and a long open reading frame encoding a single polyprotein: (NCR59)-C-prM-E-NS1-NS2A-NS2BNS3-NS4A-NS4B-NS5-(NCR39). The polyprotein is cleaved into capsid (C), precursor of membrane (prM), envelope (E) and seven non-structural proteins (NS) (20). The E protein, a major viral envelope protein, is involved in receptor binding and membrane fusion. The domain III of the E protein contains different antigenic epitopes that may be important targets for serological assays, neutralizing antibodies, and vaccines (21). Worldwide dissemination of ZIKV is thought to be due to loss of the N154 glycosylation site of the E protein, thus permitting viral adaptation to a broader range of mosquito vectors (20).

### ZIKV genotypes

Phylogenetic studies suggest that ZIKV circulating strains can be separated into two clades: African and Asiatic. The African strains are comprised of two groups: the MR766 prototype cluster (isolated in Uganda) and the Nigerian cluster. The Asian clade, on the other hand, is also comprised of two groups: the Micronesian and the Malaysian strains. ZIKV strains found in Western Africa (Ivory Coast and Senegal) were distributed between both African clusters suggesting that both lineages of the African clade are circulating in Western Africa. The NS5 and E genomic regions have the highest phylogenetic signal content and are more suitable for the reconstitution of ZIKV phylogenetic history (20). The overall genetic divergence between the African and Asiatic clades is relatively low, <11.7%. Therefore, conserved regions among the worldwide circulating ZIKV strains can be used for the design of molecular detection assays and ZIKV differentiation from other flaviviral infections. Genotyping data support the hypothesis that the genotype causing the Yap epidemic originated in Southeast Asia. It is unknown how the virus spread to Oceania. One of the hypotheses suggests that wind-blown mosquitoes can travel several hundred kilometers through the ocean and may have introduced ZIKV in islands closely situated to Southeast Asia. However, due to the great distances in the Pacific Ocean it is more likely that the virus was introduced in Oceania as a result of tourists or trade activities originating in regions endemic for the ZIKV Asian lineage (22).

### ZIKV transmission

**Figure 2:**
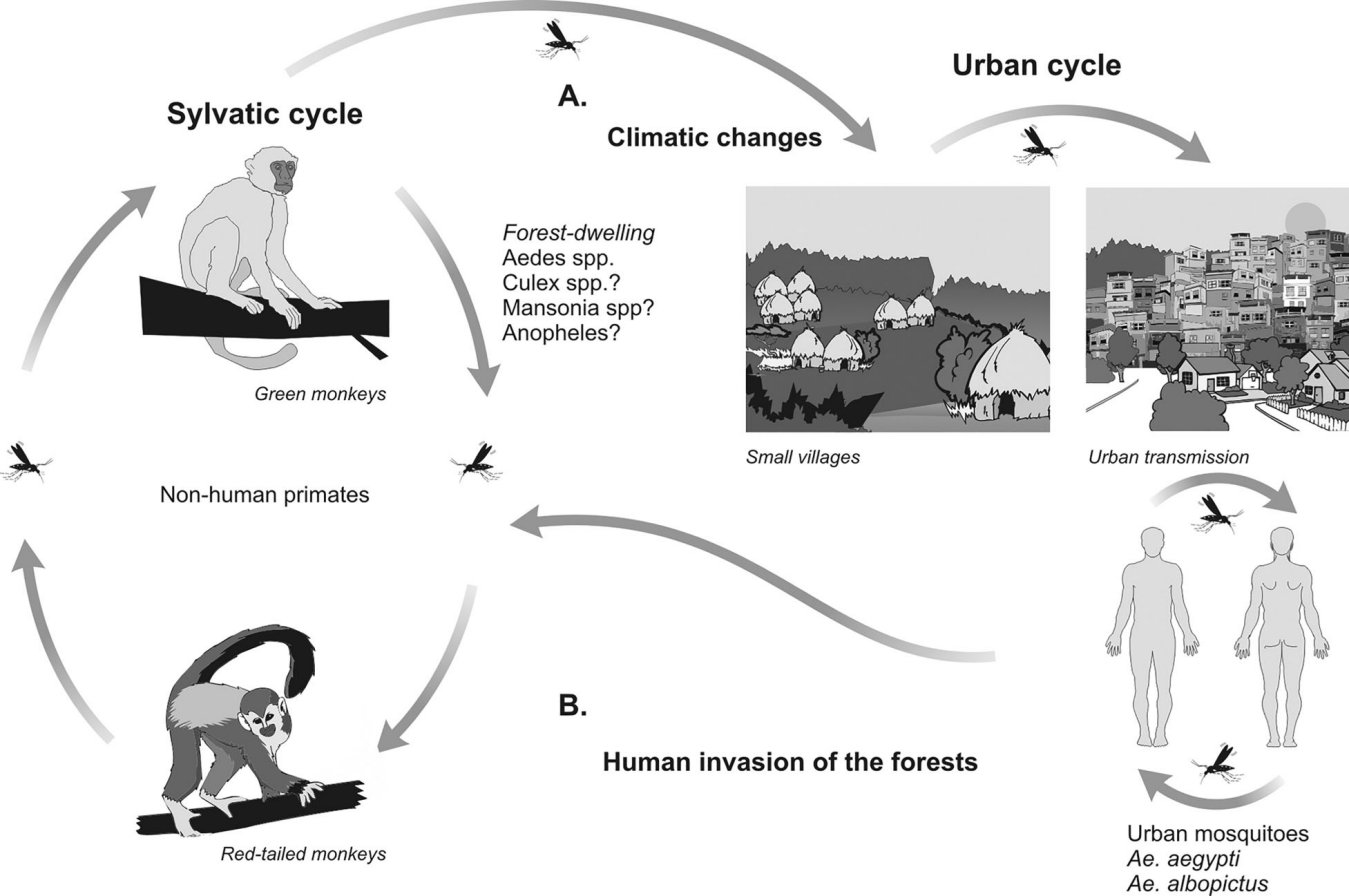
Transmission of ZIKV in its African sylvatic and urban cycles. In nature, it is thought that ZIKV infection is transmitted among non human primates with the help of different forest-dwelling mosquitoes, principally of the *Aedes* genus. It is unknown how the urban transmission occurs. Probably during heavy rainfalls, the sylvatic mosquito population can grow up progressively and disseminate the virus to nearby villages, and from there to larger urban centers, thus the urban cycle may occur with human-to-human transmission (*A*). Another possible route for human-to-human ZIKV infection is the direct human invasion of the forest habitats, where the infection can be transmitted to human hosts from forest dwelling mosquitoes (*B*).

ZIKV is an arbovirus primarily transmitted by mosquito vectors. The first isolates of ZIKV have been obtained from *Aedes (Stegomyia) africanus* (3), in Africa, and from *Aedes aegypti* (4), in the Malayan peninsula, Southeast Asia, implicating that the mosquitoes of the *Aedes* genus are the principal transmitting vectors. Consequently, ZIKV has been isolated from different *Aedes* mosquitoes in Africa including *Ae. furcifer*, *Ae. luteocephalus*, *Ae. taylori*, *Ae. dalzieli*, *Ae. opok*, *Ae. vittatus*, *Ae. jamoti*, *Ae. flavicollis*, *Ae. grahami*, *Ae. taeniarostris*, *Ae. tarsalis*, *Ae. fowleri*, *Ae. metallicus*, *Ae. minutus*, *Ae. neoafricanus, Ae. albopictus* (22-24). In Africa, ZIKV has been also isolated from mosquitoes belonging to the genera *Anopheles* (*An. coustani*, *An. gambiae*), *Mansonia* (*Ma. uniformis*) and *Culex* (*Cx. perfuscus*) (22,23). This wide range of arthropod vectors in Africa that can host ZIKV demonstrates that the virus is well adapted to different mosquito genera, which can play an important role during sylvatic and urban ZIKV cycles in Africa (Figure 2).

Despite that many mosquito species have been identified as ZIKV hosts, its reservoirs in nature remain unknown. Seroprevalence of ZIKV has been demonstrated in Old World non-human primates of the *Cercopithecus* genus (Guenons), including *Cercopithecus aethiops* (African green monkey), *Cercopithecus ascanis schmidti* (Red-tailed monkey), *Cercopithecus mona denti* (Mona monkey), and *Cercopithecus albigena johnstoni* and also the *Colobus* genus (*Colobus abyssincius*, Mantled guereza) (22). ZIKV has also been isolated from *Cerocopithecus aethiops* and *Erythrocebus patas* monkey species (22) (Figure 2). These non-human primates live in a narrow strip in Central Africa. It is possible that they are natural ZIKV reservoirs, whilst the transmission of ZIKV between them is maintained by forest-dwelling mosquitoes.

The mosquito population can proliferate during heavy rainfalls, surpass the forest boundaries, and thus ZIKV can be spread to nearby villages. Consequently, the infection can be transmitted to local inhabitants, who can participate in the dissemination of ZIKV in larger urban centers. Another possibility for ZIKV transmission is the advance of human activities in the forests, and therefore, transmission of the infection directly to humans by forest-dwelling mosquitoes (Figure 2). A recent finding demonstrated that pools of *Ae. albopictus* collected in the capital city of Gabon, Libreville, are positive for the ZIKV African lineage, strongly suggesting the participation of *Ae. albopictus* mosquitoes in the ZIKV urban cycle in Africa (25).

The epidemiologic situation of ZIKV in Asia shows some differences compared to Africa. ZIKV has been isolated from *Ae. aegypti* mosquitoes in Malaysia (4), however there is no information for another arthropod species, which can harbor ZIKV in this region. Because *Ae. aegypti* is a strict synanthropic mosquito, it is highly probable that it participates in the ZIKV urban cycles in Southeast Asia. This is additionally confirmed by the significant salivary susceptibility of urban *Ae. aegypti* captured in Singapore to the ZIKV African strain (26). The natural ZIKV reservoirs in Asia are unknown. It has been observed that orangutans (*Pongo pygmaeus*) from Borneo show high seroprevalence to different arbovirus infections, including ZIKV (27), and therefore, they may serve as ZIKV natural reservoirs in the Asiatic sylvatic cycle. *Ae. hensilli*, an abundant mosquito in the Islands of the Western Pacific Ocean, was suspected to be the predominant vector of the ZIKV Yap epidemic in 2007. However, no ZIKV RNA was detected in any of the mosquito pools collected on the island (28). Therefore, the participation of *Ae. hensilli* as transmitting vector of ZIKV needs to be elucidated. Additionally, due to the unclear epidemiology of ZIKV infection, further laboratory and field studies are crucial in order to define the viral competence for other animal and arthropod hosts, including mosquito species from newly invaded areas like the South American countries.

Other modes of ZIKV transmission, including vertical, sexual and parenteral have also been proposed. The detection of ZIKV RNA in semen from a Tahiti man complaining from hematospermia during the ZIKV outbreak in French Polynesia (29), and the probable sexual transmission of ZIKV from a man to woman with sexual contact a few days before the onset of the man's symptoms (30) suggests that the ZIKV could be transmitted sexually. The probable relationship between ZIKV and fetal microcephaly proposes parenteral transmission and risk of severe congenital infection. There is evidence for such a transmission route. Initially, during the ZIKV epidemic in French Polynesia, evidence for perinatal transmission of ZIKV has been documented (31), as judged by the detection of ZIKV RNA in plasma of newborns. Another relationship suggesting ZIKV vertical transmission is the observed unusual increase of microcephaly cases in the Northeast part of Brazil. The Brazilian health authorities are suspicious of a connection between the microcephaly and ZIKV, as the increase is associated spatio-temporally to the ZIKV outbreak in the country (32). Detection of ZIKV sequences in fetuses with malformations (microcephaly, hidrancephaly, hydrops) strongly suggests ZIKV transplacental passage and capacity to cause congenital infection with neuronal damage (33-35). However, the temporal association between ZIKV and microcephaly is not sufficient to establish a “cause-effect” relationship. The cases with confirmed microcephaly and ZIKV RNA presence are very limited compared to the total number of cases with microcephaly. Even if it is supposed that ZIKV causes intrauterine infections, more studies are needed in order to define the pathogenic effect of ZIKV on the neuronal tissues, the outcome of the infection (post-natal abnormalities) and the epidemiological burden of ZIKV in the affected countries. Moreover, an increased prevalence of microcephaly could be due to multiple causes including other viruses and parasites, irradiation, toxic substances, intrauterine growth retardation and chromosomal disorders. All of these factors have to be discarded in order to establish a vertical transmission of ZIKV and potential for induction of birth defects or stillbirths.

### Diagnosis of ZIKV

Up to this day, there is no officially approved commercial kit for ZIKV serological diagnosis. During the Yap ZIKV epidemic IgM-serological investigation on the collected samples was performed at the Arboviral Diagnostic and Reference Laboratory, Centers for Disease Control and Prevention (Atlanta, GA, USA) following a routine protocol for anti-arboviral IgM detection (36 [Bibr B37] [Bibr B38] [Bibr B39]). However, a significant proportion of the tested samples showed low levels of cross-reactivity for related flaviviruses (Japanese Encephalitis Virus, Yellow Fever Virus, West Nile Virus, Saint Louis Encephalitis Virus and Murray Valley Encephalitis Virus) and especially DENV (14). However, this result can be expected due to the complex serology of the *Flaviviridae* family resulting from the extensive cross-reactivity between the anti-flavivirus antibodies and the wide distribution of immunity against these infections in the tropical regions. The existing ZIKV serological kits on the market (Zika Virus Rapid Test, Biocan, Canada; Human Zika Virus IgG ELISA Kit, MyBioSource, USA; Anti-Zika Virus ELISA IgG/IgM, Euroimmun, Germany) are only for research purposes and their cost does not justify application during epidemics in the tropical regions. Moreover, the diagnostic specificity of these tests for samples obtained from patients living in the Tropics can be doubtful as the population of such countries demonstrates high indices of *Flavivirus* immunity.

To avoid the unspecific results obtained by ELISA, molecular detection methods can be successfully applied for ZIKV diagnosis. The results of the molecular testing are rapid and in many cases can be quantitative. Many PCR modifications with differing sensitivities are available for ZIKV detection. The most widely used assays are based on real-time hydrolysis probes (14,), however, conventional PCR methods are also available (23). ZIKV can be detected in different specimens: blood (plasma, serum), urine, saliva, and fetal tissues (14,35,40,41). A disadvantage of the PCR performed on blood samples is that the duration of ZIKV viremia is relatively short and the virus can be detected only by the time of the appearance of clinical symptoms (14). On the contrary, the ZIKV shedding in urine can be more prolonged (∼15 days after the disappearance of clinical symptoms) than the duration of viremia, and can present higher viral load. Therefore urine specimens can also be used for ZIKV confirmation (40,42,43). Moreover, the collection of urine is a non-invasive procedure that makes this specimen interesting for ZIKV diagnosis, especially in children and newborns. During the ZIKV outbreak in French Polynesia the virus has been detected in saliva (41), which can also be used for ZIKV detection. However, it is unknown how long the liberation of viral particles in saliva lasts. Manipulation with this type of specimen can be tricky due to its insufficient volume and usually low viral quantities. We believe that for confirmation of ZIKV infection a combined approach must be applied including blood testing as a principal specimen for ZIKV RNA detection, and urine/saliva testing as additional markers for viral confirmation. On the contrary, for the confirmation of congenital ZIKV infection and the relationship of ZIKV with fetal abnormalities, the molecular test must be performed on fetal tissues (brain, placenta) (35,44) or amniotic fluid (34).

For ZIKV confirmation, viral isolation on cell cultures is also possible. However, ZIKV isolation in cell cultures can be time consuming and is not suitable for routine diagnosis. ZIKV is best isolated after intracerebral inoculation of clinical materials into newborn mice (1). ZIKV can also be isolated on different cell lines including mosquito-derived (AP-61, *Ae. pseudoscutellaris;* C6/36, *Ae. albopictus*) or derived from non-human primates (VERO, kidney epithelial cells from African green monkey; BHK, baby hamster kidney cells) (22).

The diagnosis of ZIKV infection seems to be a complex issue. The principle sample for virus RNA detection can be plasma, however, it must be combined with the simultaneous ZIKV PCR on urine/saliva. For confirmation of congenital infection, PCR on amniotic fluid is plausible. Due to the short duration of the viremia, specimens must be collected immediately during the onset of the clinical disease. ZIKA serological confirmation is possible, despite its limited diagnostic significance and high cost.

### ZIKV clinical manifestations and treatment

Although ZIKV infection is thought to be asymptomatic in approximately 80% of infected people, the virus causes a mild, non-specific and self-limiting infection resembling Chikungunya or DENV fevers (21). The duration of the incubation period is thought to last a few days. The classic clinical picture of ZIKV infection is manifested by low-grade fever (37.8 to 38.5°C), bilateral non-purulent conjunctivitis, headache, myalgia and arthralgia with periarticular edema of the small joints lasting for up to one week. ZIKV infection also causes a generalized, erythematous, maculopapular rash that spreads from the face to the limbs (21).

Patients with uncomplicated ZIKV disease are generally not treated, since the symptoms resolve within 3-7 days (21). The only available treatment for the infection is palliative. Fever and/or arthralgia can be treated with acetaminophen and the itching with anti-histamine drugs. It is also recommended an adequate rehydration due to fluid loss. Aspirin and nonsteroidal anti-inflammatory medicaments are not recommended due to the possibility of misdiagnosing the infection as DENV (21).

Despite the ZIKV classic mild illness, it is thought that this virus can also exert a pathological effect on neuronal tissues of a fetus or an adult. During the French Polynesia ZIKV outbreak (2013-2014), a 20-fold increase of Guillain-Barré syndrome (GBS) was reported compared to previous years (32). Another evidence for ZIKV neurotropism is the reported case of a young girl with acute myelitis with a possible ZIKV etiology. Moreover, during the Brazilian ZIKV outbreak, the first associations of ZIKV and fetal neuronal malformations were reported. Although, no causal relationship between ZIKV infection and neurological damage has been established yet, there is significant evidence supporting such a hypothesis.

### Neurological non-congenital ZIKV effect

GBS is characterized by progressive paralysis over 1-3 weeks due to an immune response, typically occurring after minor viral or bacterial infections. These include influenza, *Campylobacter jejuni,* cytomegalovirus and Epstein-Barr virus (45-47). Patients usually present affected motor function, beginning distally and progressing proximally. This includes bilateral weakness of the arms and legs, areflexia, sensory disturbances and involvement of the cranial nerves and muscles, in some cases affecting the eye movements or swallowing (48). These symptoms can last from few weeks to several months. The risk of GBS increases with age, and men are more commonly affected than women (49). The clinical outcome of GBS is related to a 5% death rate and 20% of the patients remain with significant disability (50).

The recent outbreaks of ZIKV demonstrated that this virus can be involved in the development of GBS. The first evidence of ZIKV-associated GBS was reported during the French Polynesia outbreak (51), in which 42 patients were diagnosed with subacute flaccid paralysis after ZIKV infection (52). Most of them (88%) had shown classical ZIKV symptoms with onset of the neurological disease in about 6 days. The patients presented generalized muscle weakness, incapacity to walk, facial palsy and increased protein concentration in the cerebrospinal fluid (CSF). None of them demonstrated ZIKV RNA in the serum but presented anti-ZIKV IgM (93%), IgG (69%), and neutralizing antibodies (100%) (52).

The treatment for ZIKV-induced GBS is only palliative. Approaches include application of intravenous immune globulins and in rare cases, plasmapheresis. The patients showed rapid recovery, and after 3 months 57% of them were able to walk without assistance. The GBS incidence during the French Polynesia ZIKV outbreak was estimated to be approximately 0.24% ZIKV infections (52).

Another neurological commitment recently related to ZIKV is the acute myelitis (inflammation of white/gray matter of the spinal cord). A patient from the Guadalupe Island presented a clinical picture of myelitis and severe pain. High ZIKV RNA load was detected in serum, urine and CSF nine days after symptoms onset. No other infectious agent was detected. The patient was treated with methylprednisolone and after 1 month the legs were still presenting moderate weakness (53). No relationship between myelitis and ZIKV infection has been established. However, the neurotropism of some flaviviruses such as DENV (54), Japanese encephalitis (55), and West Nile (56) causing encephalitis and myelitis is well documented, therefore, it is highly probable that ZIKV can also be involved as a causing agent in inflammatory neurological disease.

### Congenital ZIKV infection

ZIKV infection has become an important worldwide public health issue since its association with microcephaly in neonates in Brazil (57).

Microcephaly is characterized by a smaller occipital frontal circumference of the head of any newborn, than expected for age, evident at birth (primary microcephaly) or postnatally (secondary microcephaly). There is no consensus in the scientific community for the diagnosis of fetal microcephaly, especially in the context of ZIKV infection (58). In general, during prenatal observations the measure of the occipital frontal circumference by ultrasound is compared to the fetal age-related mean, in order to establish how small it is in relation to the gestational age. There are different causes for microcephaly development, such as exposure to environmental factors (heavy metals, smoking, alcohol, radiation), genetic causes, maternal diabetes, and pathogens (cytomegalovirus, toxoplasmosis, rubella, herpes simplex, syphilis, HIV, and West Nile virus) (32). More recently, ZIKV was also associated with development of microcephaly (57).

According to the WHO, a newborn with head circumference equal to or lower than two standard deviations (≤2 SD) below the mean is characterized as microcephalic, and below 3SD is referred to as severe prognosis of microcephaly (59). The clinical findings in microcephaly include marked cerebral atrophy and ventriculomegaly, extensive intracranial calcifications, simplified gyral patterns, dysgenenis of the corpus collosum, and cerebellar hypoplasia (60). Consequently, it can be associated with intellectual disability, developmental delay and seizures. Microcephaly is a lifelong condition and there is no cure or standard treatment. However, it is worth noting that most fetuses diagnosed prenatally by ultrasound will not have pathological microcephaly at birth, despite their small head size.

A report from Paraiba, Brazil related two fetuses with microcephaly by prenatal ultrasound from two pregnant women who had ZIKV symptoms but were ZIKV RNA negative in blood, and ZIKV positive in amniotic fluid. One of the neonates was born with 40 weeks gestational age and head circumference almost 3SD below the mean. The other neonate was born with severe ventriculomegaly, microphtalmia, cataract and severe arthrogryposis of the legs and arms (61). Similar results were obtained by the study of Mlakar et al. (2016), where ZIKV positive fetus with microcephaly had almost complete agyria, internal hydrocephalus, and calcifications in the cortex and subcortical white matter in the frontal, parietal, and occipital lobes (35). Almost all of the case-reports demonstrate fetal abnormalities apart from microcephaly including multifocal intracranial and placental calcifications, ventriculomegaly, intrauterine growth restriction, brain atrophy, and other less frequent abnormalities (21,35,44,58,62,63). Fetal abnormalities can be detected in up to 29% of ZIKV-positive pregnant women (62). Preliminary analyses demonstrate that the highest risk of microcephaly or congenital anomalies is during the first trimester of pregnancy (57,58), however, ZIKV congenital abnormalities were observed in fetuses of women who were infected by ZIKV at any week of gestation (62). The severity of other neurological damages associated with congenital infections like rubella e cytomegalovirus is inversely related to the gestational period of the fetal infection (58). Thus, more data is needed to correlate fetal abnormalities to time of infection, once it could be related with severe sequelae in the newborn.

Fetal deaths and miscarriages have also been reported in ZIKV-positive pregnant women. The reported miscarriages occurred during the first trimester (11th and 13th weeks) (44,62). Women who presented fetal deaths were infected with ZIKV during the second and third trimester, and presented at least maculopapular rash during the acute phase of infection (62). Newborn lethality within the first 20 h after birth from ZIKV infected mothers has also been reported (44).

Congenital ocular findings concomitant with microcephaly have also been associated with ZIKV infection during pregnancy. Ocular abnormalities were present in 34.5% of microcephalic infants examined, and involved the bilateral vision in 70% of them. The lesions included focal pigment mottling of the retina, chorioretinal athropy, optic nerve abnormalities, bilateral iris coloboma (congenital fissure) and lens dislocation. These lesions are considered vision threatening eye damage and also can be caused by West Nile virus infection, toxoplasmosis, cytomegalovirus, rubella, herpes simplex virus and syphilis. This study shows the importance of testing microcephalic newborns for ocular abnormalities, since the rate is reported to be very high during the ZIKV epidemic in Brazil (64).

Other tissues besides neuronal and optical may also be affected by ZIKV congenital infection. This association was demonstrated in a report from Salvador, Brazil, which showed a fetus miscarriage (32nd week) with microcephaly, hydranencephaly, intracranial calcifications and destructive lesions of posterior fossa. Hydrotorax, ascites and subcutaneous edema were also reported. It is suggested that the mother had an asymptomatic ZIKV infection during the 1st trimester of gestation. ZIKV RNA was detected at cerebral cortex, medulla oblongata, cerebrospinal and amniotic fluid. Other organs did not present viral RNA (33).

### Lessons learned from the ZIKV epidemic in Brazil

Currently, Brazil is experiencing the largest ZIKV epidemic among the countries of Latin America. Preliminary data announced on December 2015, by the European Centre for Disease Prevention and Control, estimate that between 440,000 and 1.3 million cases of autochthonous transmission of ZIKV have occurred in Brazil, but these data are largely underestimated and the real magnitude of the Brazilian ZIKV epidemic may be much higher (59).

The most significant problem in Brazil, concerning the ZIKV epidemic is the relationship between the virus and development of fetal abnormalities. The observations began in September 2015, when an increasing number of newborns with microcephaly was reported from the relatively small Pernambuco State, Northeast Brazil. This preliminary data demonstrated significant increase of this condition when compared to previous years and the governmental authorities started a national task force program for defining the cause. The first official report suggesting a relationship between ZIKV and congenital neurological malformations for the period between August-October 2015 was published early in 2016 (57). Consequently, ophthalmological abnormalities like macular atrophy and cerebral calcifications were attributed to ZIKV infection acquired during pregnancy, but without ZIKV RNA detection (65). The first possible relationship between ZIKV and development of microcephaly was reported for a pregnant Slovenian woman, who lived temporarily in Brazil and experienced febrile illness with rash at the end of the first trimester of pregnancy. The ultrasonography performed at 29 weeks of gestation revealed severely affected fetal central nervous system with calcifications and gross intrauterine growth retardation. In the aborted fetus, ZIKV RNA was detected uniquely in fetal brain tissues with high viral load (6.5×10^7^ copies/mg tissue) and the complete genome of the infected strain was sequenced. The phylogenetic analysis showed close resemblance to the Asian clade. More recently, studies performed in Brazil found a more direct relationship between ZIKV infection and microcephaly as confirmed by the detection of ZIKV genome in the amniotic fluid of pregnant women (34) and in newborns with severe birth defects (33). A prospective study performed between September, 2015 and February, 2016 in pregnant women who tested positive for ZIKV in blood/urine demonstrated fetal abnormalities in 12 of 42 fetuses (62). Moreover, a study examining ZIKV effect on human neural progenitor cells demonstrated that the infection increases cell death and dysregulates cell-cycle progression resulting in attenuated cell growth (66). These results suggest that ZIKV can be involved in the development of congenital infection, which can be responsible for the appearance of fetal abnormalities, especially in the central nervous system. Up to February 27, 2016 according to the Brazilian Ministry of Health, 5,640 cases of microcephaly in the country have been reported, and 583 of them are confirmed to have microcephaly and/or other central nervous system findings, suggestive of congenital infection. Of the total cases with confirmed microcephaly, only 67 fulfill the laboratory criteria for ZIKV infection, and 4,107 across Brazil remain under investigation (67). Although, there has been evidence that ZIKV can cause severe congenital infection, many questions remain unanswered. The most important are, how the virus reaches the fetus, what is the ZIKV impact during the different periods of formation of the fetus, and if the virus is detected long after acute infection in fetal brain tissues, what are the sites of viral persistence in the fetus.

The burden of microcephaly cases in Brazil poses serious problems for the health authorities, not only due to the high cost of the medical care involved in the assistance of the families, but also to the fact that the majority of the cases is clustered in the poorest regions of Brazil with precarious healthcare. The difficulty arises also from the difficulty in controlling the population of the presumable transmitting vector *Ae. aegypti* in the extensive national territory of Brazil, despite the involvement of the Brazilian Army. Despite the measures taken, the epidemic potential of ZIKV shows that Brazil is not prepared to deal with large urban outbreaks of tropical viral diseases, and organized efforts are necessary not only to control the vectors population, but also to educate the people about the clinical impact of the arboviral diseases. More investments are also necessary to demonstrate the scientific link between ZIKV infection and development of fetal neuronal abnormalities, and to develop a vaccine for the immunization of the general population.

## Conclusion

The emergence of ZIKV in Brazil with the involvement of a large number of cases shows that arboviral infections can easily cross international frontiers and have serious impact in countries that have no preparedness or programs to deal with extensive outbreaks. Moreover, the probable relationship between ZIKV and congenital microcephaly in Brazil poses a great burden at the national health system of the country, which would have to be involved in the support of the affected children for their lifetime. Despite the fragmented reports of various Brazilian scientific research groups regarding the pathogenesis of ZIKV infection, there is no scientific unification to study in more details the transmission, the clinical impact and the possibility of treatment of this flaviviral infection. Strategies for the development of a vaccine are also imperative. Similarly, a serological test to perform a robust serologic survey throughout the national territory to evaluate the impact and epidemiology of ZIKV is urgently needed. Better understanding of the natural history of ZIKV infection is needed in order to establish effective control measures for this outbreak in Brazil and Latin America.
